# Surface Wrinkled Microsphere Enhanced Irregular Wound Healing Through Synergistic Hygroscopicity, Reversible Wet‐Adhesion and Antibacterial Properties

**DOI:** 10.1002/smsc.202300216

**Published:** 2024-01-03

**Authors:** Zhan Xu, Yuqian Cui, Weiguo Tian, Feifei Sun, Jun Zhang

**Affiliations:** ^1^ Beijing National Laboratory for Molecular Sciences CAS Key Laboratory of Engineering Plastics Institute of Chemistry Chinese Academy of Sciences (CAS) Beijing 100190 China; ^2^ School of Chemical Sciences University of Chinese Academy of Sciences Beijing 100049 China; ^3^ CAS Key Laboratory for Biomedical Effects of Nanomaterials & Nanosafety CAS Center for Excellence in Nanoscience National Center for Nanoscience and Technology Beijing 100190 China

**Keywords:** antibacterial, dressing, hygroscopicity, microsphere, wound healing

## Abstract

Rapid and effective healing of irregular wounds caused by burns, lacerations, and blast injuries remains a persistent challenge in wound care. Hydrogel microsphere dressings that can adaptively fill and adhere to the wounds without external force are desired to treat irregular wounds, providing an external barrier and accelerating healing. Herein, we created multifunctional cellulose‐based surface‐wrinkled microspheres with antioxidant, antibacterial, hygroscopicity, wet‐adhesion and shape‐adaptive capabilities to relieve inflammation, bacteria and excess exudate situations in healing irregular wounds. This dressing rapidly adsorbs exudate and reversibly adheres wetly to the wounds upon being filled, effectively inhibiting bacterial infection and reducing the flooded exudate to accelerate wound healing. Polydopamine (PDA) provides catechol‐based tissue bioadhesion to microspheres through *π*–*π* stacking or hydrogen bond interaction, and also establishes a bond bridge with an antimicrobial component (thymol), which not only enables the microspheres to stably adhere to the wound to maintain hygroscopicity, but also improves the release of the introduced antimicrobial component (thymol). In vivo assays, as well as histopathological and immunofluorescence studies have shown that multifunctional cellulose‐based microspheres have excellent pro‐healing abilities and are promising candidates for dehumidification and healing of irregular wound in clinical applications.

## Graphical abstract

Multifunctional cellulose‐based surface‐wrinkled microspheres with antioxidant, antibacterial, hygroscopicity, wet‐adhesion and shape‐adaptive capabilities that can adsorb exudate and adhere to the wounds, therefore, effectively inhibit bacterial infection, relieve inflammation, and reduce the flooded exudate to accelerate wound healing, and are promising candidates for dehumidification and healing of irregular wound in clinical applications.

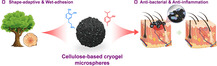

## 1 Introduction

Rapid and effective healing of irregular wounds caused by burns, lacerations, and blast injuries has been a long‐term challenge in clinical wound care.^[^ ^1^, ^2^, ^3^, ^4^, ^5^ ^]^ Irregular wounds require dressings that can be adaptively filled or wet adhered to the wound for effective treatments, as they provide flexible antimicrobial barrier to promote healing.^[^ ^6^, ^7^, ^8^, ^9^ ^]^ Generally, traditional wound dressings (e.g., gauze, sponge, membrane, and hydrogel) have limited exudate absorption ability, and their fixed shape makes it challenging to maintain complete contact with irregular wounds.^[^ ^10^, ^11^ ^]^ These limitations leave parts of the wound exposed to the external environment and excessive deposition of exudate.^[^ ^12^, ^13^ ^]^ Excessive deposition of tissue exudates can trigger bacterial proliferation,induce an inflammatory response and reactive oxide species (ROS) accumulation, which thereby delays healing.^[^ ^14^, ^15^, ^16^ ^]^ Conventional wound dressings often need to be pre‐prepared in a specific shape to treat irregular wounds.^[^ ^17^, ^18^, ^19^ ^]^ However, this can delay treatment and impose an additional financial burden. Recently, researchers have reported in situ‐formed wound dressings such as injectable hydrogels to circumvent the drawbacks of conventional dressings for irregular wounds.^[^ ^20^, ^21^, ^22^ ^]^ Nonetheless, long gelation time, poor mechanical properties, and lack of an extracellular matrix‐like (ECM) structure of these dressings have limited their potential for clinical application.^[^ ^23^, ^24^, ^25^ ^]^ In addition, the gelation process of most of injectable hydrogels need to be accomplished under invasive external stimuli, such as pH, light, temperature or organic cross‐linked agents.^[^ ^26^, ^27^ ^]^ To overcome these limitations, it is essential to develop wound dressings that can fill wounds without external force and have antibacterial and antioxidant properties.

A dressing must prevent bacterial infection and eliminate inflammation in the early stages of injury (inflammatory phase) as well as provide a long‐term, stable sterile microenvironment to promote wound healing.^[^ ^28^, ^29^, ^30^ ^]^ Many reports have described successful incorporation of antibacterial agents (e.g., organic) into dressings to accelerate wound healing. Notably, organic phenolics have excellent antibacterial and antioxidant abilities, which are expected to simultaneously resolve the challenges of bacterial infection and inflammation‐driven ROS accumulation.^[^ ^31^, ^32^, ^33^ ^]^ However, overuse of organic antibacterial drugs leads to bacterial resistance and reduces therapeutic effect on the wounds.^[^ ^34^, ^35^, ^36^ ^]^ Mussel‐inspired materials exhibit excellent antimicrobial efficacy against some strains (e.g., *Escherichia coli*, *Pseudomonas aeruginosa*, and *Staphylococcus aureu*s) via a contact‐active antimicrobial mechanism, allowing them to be used as a secondary antimicrobial component in wound dressings to provide long‐term, stable protection.^[^ ^37^, ^38^, ^39^ ^]^ A universal and effective wound‐healing strategy is presented here which includes a slight amount of phenolic antimicrobial agents (short‐term antimicrobial agents) and mussel‐inspired material (long‐term antimicrobial agents) in dressings to prevent bacterial infection, oxidative stress and accelerate wound healing.

We developed a multifunctional cellulose‐based microsphere with water‐absorbent, antibacterial, antioxidant, and shape‐adaptive properties to accelerate healing of irregular wounds. The surface‐wrinkled and porous microspheres with uniform size (400–450 μm), high porosity, and 3D hydrophilic network can rapidly absorb exudate and perfectly adhere to irregular wounds to inhibit bacterial growth and promote wound healing. The introduction of polydopamine (PDA) by in‐situ polymerization provides catechol‐based tissue adhesion to ensure that the microspheres stably adhere to the wound surface. Moreover, due to its *π*–*π* stacking or hydrogen bonding interactions, PDA coating serves as a bridge for secondary reactions with other compounds, which can adsorb more phenolic antimicrobials (thymol [Tm]), inhibiting wound infection in the inflammatory phase of healing and reducing the ROS accumulation. In vitro antibacterial tests showed that the antibacterial capacity in the inflammatory phase was mainly provided by Tm, and after the rapid and complete release of Tm, the PDA coating provided enduring antibacterial and wound protection. The efficacy of the cellulose‐based microspheres was evaluated in a rat model of full‐thickness skin wounds, in which wound healing was accelerated by promoting granulation tissue and neovascularization. Thus, surface‐wrinkled cellulose‐based microspheres combined with excellent antibacterial (long, short term), antioxidant, and shape‐adaptive properties show great potential for irregular wound‐healing applications.

## 2 Results and Discussion

### 2.1 Preparation and Properties of the Cellulose‐Based Cryogel Microspheres

**Figure 1:**
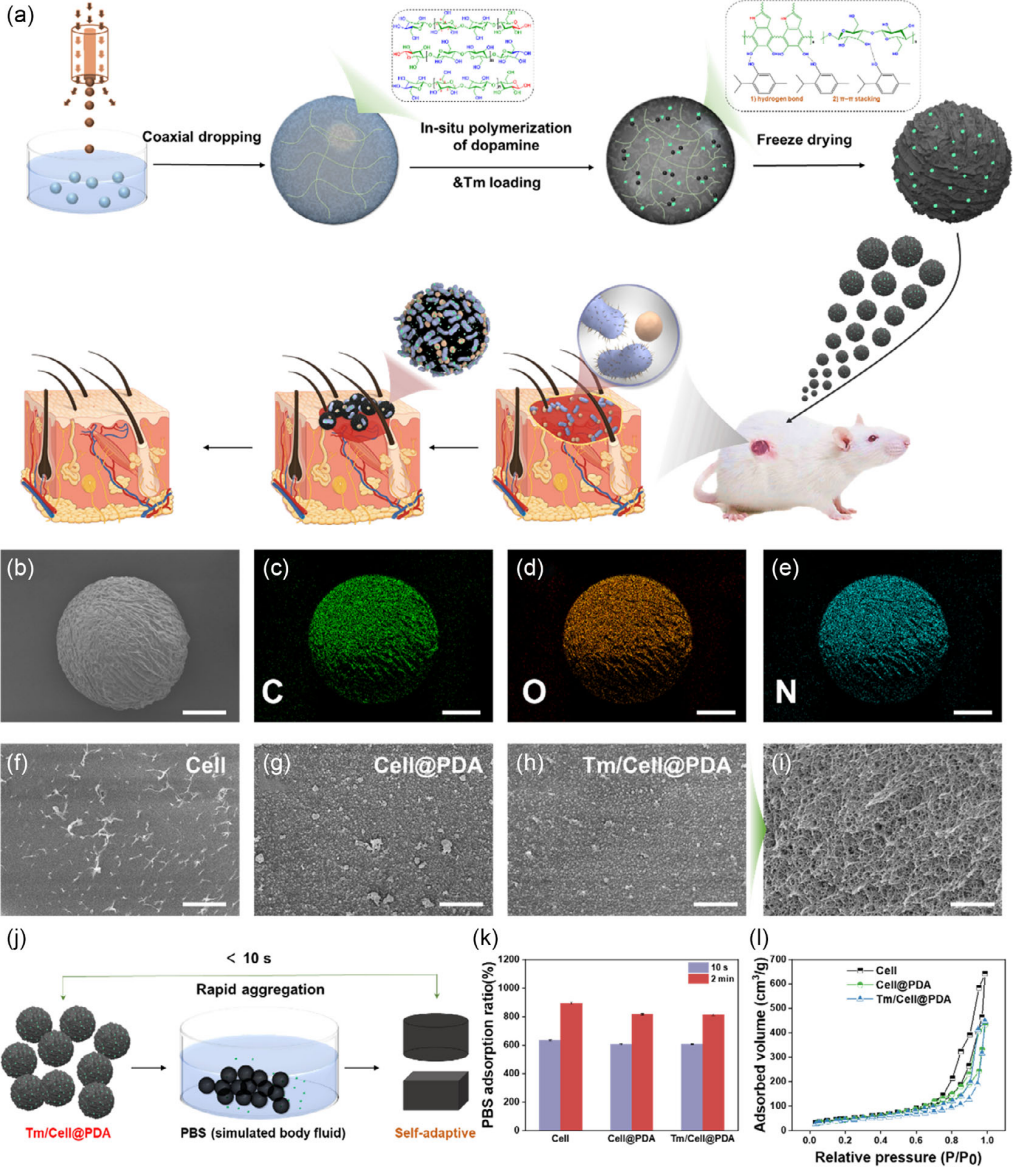
Wound‐adaptive dressings based on cellulose‐based cryogel microspheres (Tm/Cell@PDA) and their fundamental properties. a) Schematic illustration of the preparation process of Tm/Cell@PDA for irregular wound healing. b–e) SEM and EDS images of Tm/Cell@PDA cryogel microspheres, scale bar, 100 μm. Surface micromorphologies of cellulose‐based cryogel microspheres: f) Cell, g) Cell@PDA, h) Tm/Cell@PDA. Scale bar, 1 μm. i) Cross‐section SEM image of Tm/Cell@PDA, scale bar, 1 μm. j) Schematic diagram of the process of measuring the PBS absorption of Tm/Cell@PDA and the formation of wound‐adapted wet hydrogels. k,l) PBS absorption ratio (*n* = 4) and nitrogen absorption−desorption isotherms of cryogel microspheres (Cell, Cell@PDA, Tm/Cell@PDA). All data are presented as mean ± standard deviation (SD) according to duplicated experiments more than 3 times.

To address the current challenges of irregular wound treatment, we designed cellulose‐based multifunctional cryogel microspheres with antibacterial, antioxidant, and shape‐adaptive properties. The microspheres were prepared by a three‐step process (**Figure** **1**a). First, the cellulose/AmimCl solution was dispersed into homogeneous droplets via the coaxial airflow method, and the droplets were flown into the coagulating bath (ethanol) to obtain cellulose hydrogel microspheres (Cell‐hm). Then, PDA was introduced on the Cell‐hm surface, and cellulose hydrogel microspheres wrapped with PDA (Cell@PDA‐hm) were obtained via in situ polymerization. The antimicrobial and antioxidant component Tm was doped via adsorption to cellulose (hydrogen bonding) or PDA (*π*–*π* stacking and hydrogen bonding), yielding Tm/Cell@PDA‐hm microspheres. Finally, the microspheres (Cell‐hm, Cell@PDA‐hm, and Tm/Cell@PDA‐hm) were freeze‐dried to obtain cellulose‐based cryogel microspheres (Cell, Cell@PDA, and Tm/Cell@PDA) with highly wrinkled surfaces, high porosity and uniform size (400–450 μm, Figure 1b and S1, Supporting Information) for irregular‐wound healing. The microspheres absorb exudate and rapidly release Tm while filling wounds to prevent initial infection. The PDA coating provides long‐term bacterial growth inhibition to promote wound healing after the complete release of Tm.

The chemical composition and microstructure of cellulose‐based multifunctional cryogel microspheres (Tm/Cell@PDA) enhance its potential for accelerating wound healing. The distribution of C, N, and O on the surface of Tm/Cell@PDA was assessed by in‐situ EDS testing (Figure 1c–e), and the uniform distribution of N elements indicated that a large amount of PDA existed on Tm/Cell@PDA. In contrast, the Cell surface is relatively smooth, and uniformly scattered PDA nanoparticles were observed on the surface of Cell@PDA and Tm/Cell@PDA (Figure 1f–h). The successful introduction of Tm was confirmed by XRD (Figure S2, Supporting Information), which showed the characteristic curves of Cell and Cell@PDA almost overlap, and only typical diffraction peaks belonging to cellulose II crystal can be observed (12.3°, 20.1°, and 34.7°, corresponding to 110, 110/020, and 004 crystallographic planes). In contrast, the XRD profile of Tm/Cell@PDA showed a sharp Tm crystalline peak in addition to the characteristic peak of Cell. Similar results are observed in FTIR characterizations. As seen in Figure S3a, Supporting Information, the peaks at 2958 and 2869 cm^−1^ are attributed to CH_3_ (stretching) in Tm. The peaks from 1600 to 1510 cm^−1^ are assigned to the phenol ring of Tm, while the strong peaks at 944, 854, 806, and 738 cm^−1^ are related to aromatic C─H (waging vibration). These results indicate that Tm has been doped in the cellulose‐based microspheres. Moreover, PDA in cellulose microspheres can be confirmed by the characteristic N1 peaks (400.0 eV) shown in the XPS spectra of Cell@PDA and Tm/Cell@PDA (Figure S3b, Supporting Information), and these characteristic peaks are absent from the XPS plot of Cell.

The microspheres (Tm/Cell@PDA) are small, highly water absorbent, and provide excellent tissue adhesion to absorb wound exudate and allow Tm/Cell@PDA to stably adhere to the wound, ensuring Tm/Cell@PDA fully fill with the irregular wound and complete release of antibacterial and antioxidant components (Tm) at the wound site. As shown in the cross‐sectional SEM of Tm/Cell@PDA (Figure 1i), Tm/Cell@PDA have an interpenetrating three‐dimensional nanopore structure and extremely high specific surface area, both of which were confirmed by the nitrogen adsorption–desorption curves (Figure 1l). The high specific surface area facilitates the uptake of tissue fluid, which otherwise provides a rich breeding microenvironment for bacterial infection. Tm/Cell@PDA can rapidly absorb a large amount of PBS over 6 times their own weight in 10 s and closely aggregated together to form a wound‐adaptive barrier to seal the wound without applying pressure (Figure 1j,l). Moreover, the PBS adsorption ratios of Tm/Cell@PDA and Cell@PDA were still slightly lower than Cell with 2 min of adsorption (Figure 1k). This slight difference in adsorption ratio may be because PDA blocks some of the internal pores of the microspheres, and this result is also consistent with that conclusion in Figure 1l and Table S1, Supporting Information.

### 2.2 Evaluation of the Release Kinetics of Tm in Cellulose‐Based Microspheres

**Figure 2:**
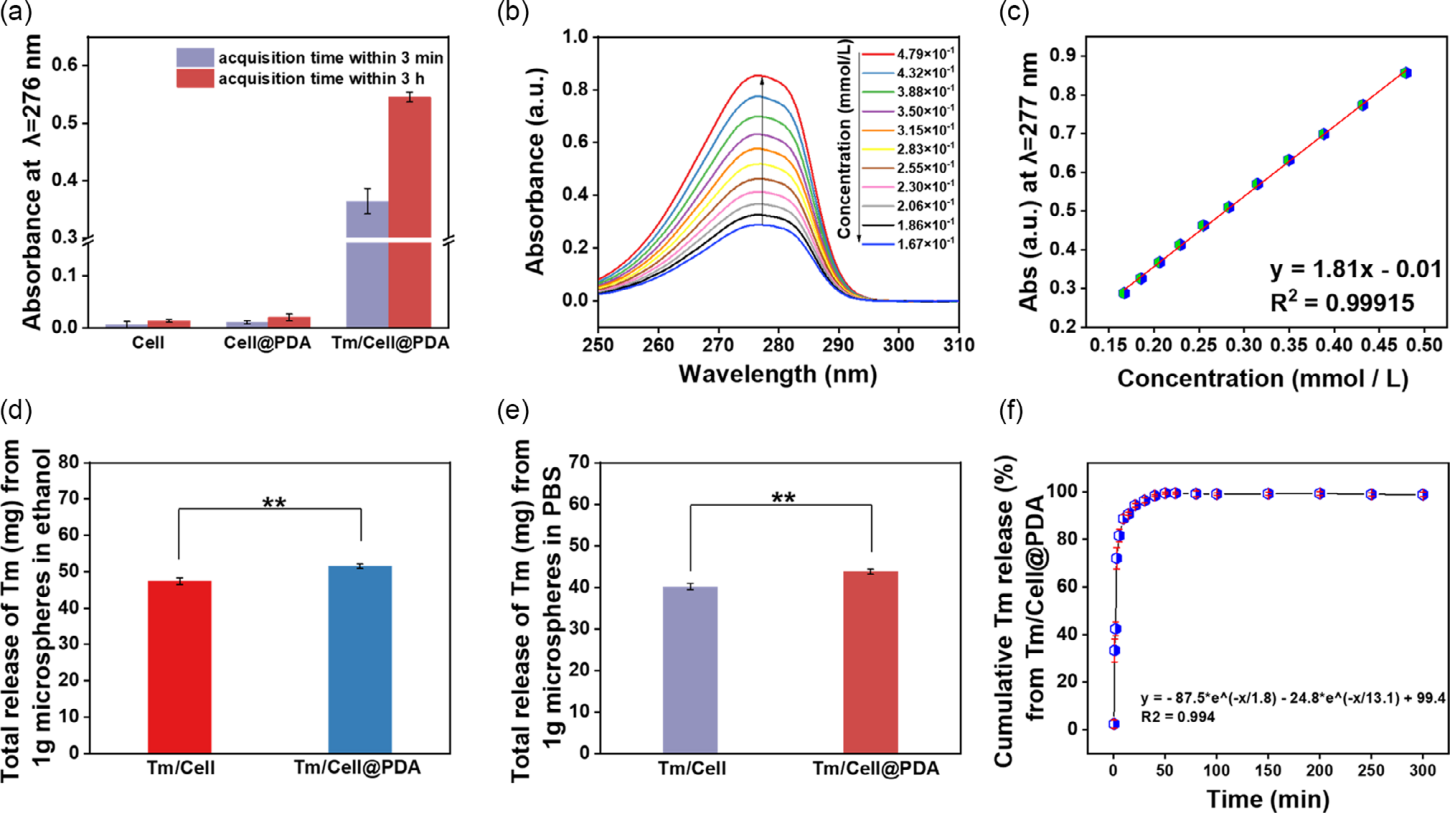
Evaluation of the release kinetics of cellulose‐based microspheres. a) UV‐vis spectra of the PBS solutions of Cell, Cell@PDA, and Tm/Cell@PDA cryogel microspheres with different soaking time (*n* = 3). b) UV‐vis spectra of thymol/ethanol solutions at different concentrations (wt%). c) Calibration plot of concentration vs absorbance. Total release of thymol from 1 g of Tm/Cell@PDA cryogel microspheres in d) ethanol (*n* = 3) and e) PBS (*n* = 3). f) Cumulative release curve of thymol absorbed in Tm/Cell@PDA cryogel microspheres (*n* = 3). All data are presented as mean ± standard deviation (SD) according to duplicated experiments more than 3 times.

Tm exerts antibacterial and antioxidant effects during the inflammatory phase of trauma, and its rapid release can scavenge ROS and prevent bacterial infection, thus accelerating wound healing. We investigated the Tm release kinetics from Tm/Cell@PDA using a calibration curve. First, we tested the UV‐vis spectra of the PBS dispersions of Cell, Cell@PDA, and Tm/Cell@PDA, and found that only Tm/Cell@PDA showed a strong absorption peak at 276 nm (**Figure** **2**a and S4, Supporting Information), demonstrating successful incorporation of Tm into the microspheres. Release of Tm was also assessed by absorption at 276 nm, which showed a linear relationship between absorption values and concentration (0.015–0.05 mmol L^−1^) (Figure 2b,c).

By separately investigating the release behavior of Tm/Cell and Tm/Cell@PDA in different solvents (e.g., ethanol and PBS) (Figure 2d,e), we found that Tm in Tm/Cell@PDA (total amount, 51.5 ± 0.6 mg; actual release, 43.8 ± 0.6 mg) was greater than in Tm/Cell (total amount, 47.4 ± 1.0 mg, *p* < 0.01; actual release, 40.2 ± 0.7 mg, *p* < 0.01), although the specific surface areas were discordant, perhaps because the *π*–*π* stacking and hydrogen bonding interactions of PDA can also assist in Tm uptake. The higher content of Tm ensures the avoidance of wound infection and the removal of excess ROS during the early stages of healing. Nevertheless, 1 g of Tm/Cell@PDA contains only about 50 mg of Tm, which delay the generation of bacterial resistance as much as possible. We explored the release kinetics of Tm in simulated body fluid (PBS) using Tm/Cell@PDA as a model (Figure 2f). Due to rapid liquid absorption by Tm/Cell@PDA, PBS diffused into the internal structure of Tm/Cell@PDA, and the microspheres swell rapidly, relaxing and allowing release of Tm in a first‐order kinetic process (*R* ^2^ > 0.994)

(1) $$\text{Cumulative Tm release }\left(\%\right)=-87.5e^{\left(-t/1.8\right)}-24.8e^{\left(-t/13.1\right)}+99.4$$

Tm release occurs quickly at the beginning, reaching 88.7–90.5% at 10 min, after which the rate levels off and the release amount reaches equilibrium by 60 min, thus providing rapid drug delivery in the inflammatory phase, limiting bacterial reproduction and providing a sterile environment for healing.

### 2.3 In Vitro Biological and Antioxidant Evaluation of Cellulose‐Based Microspheres

**Figure 3:**
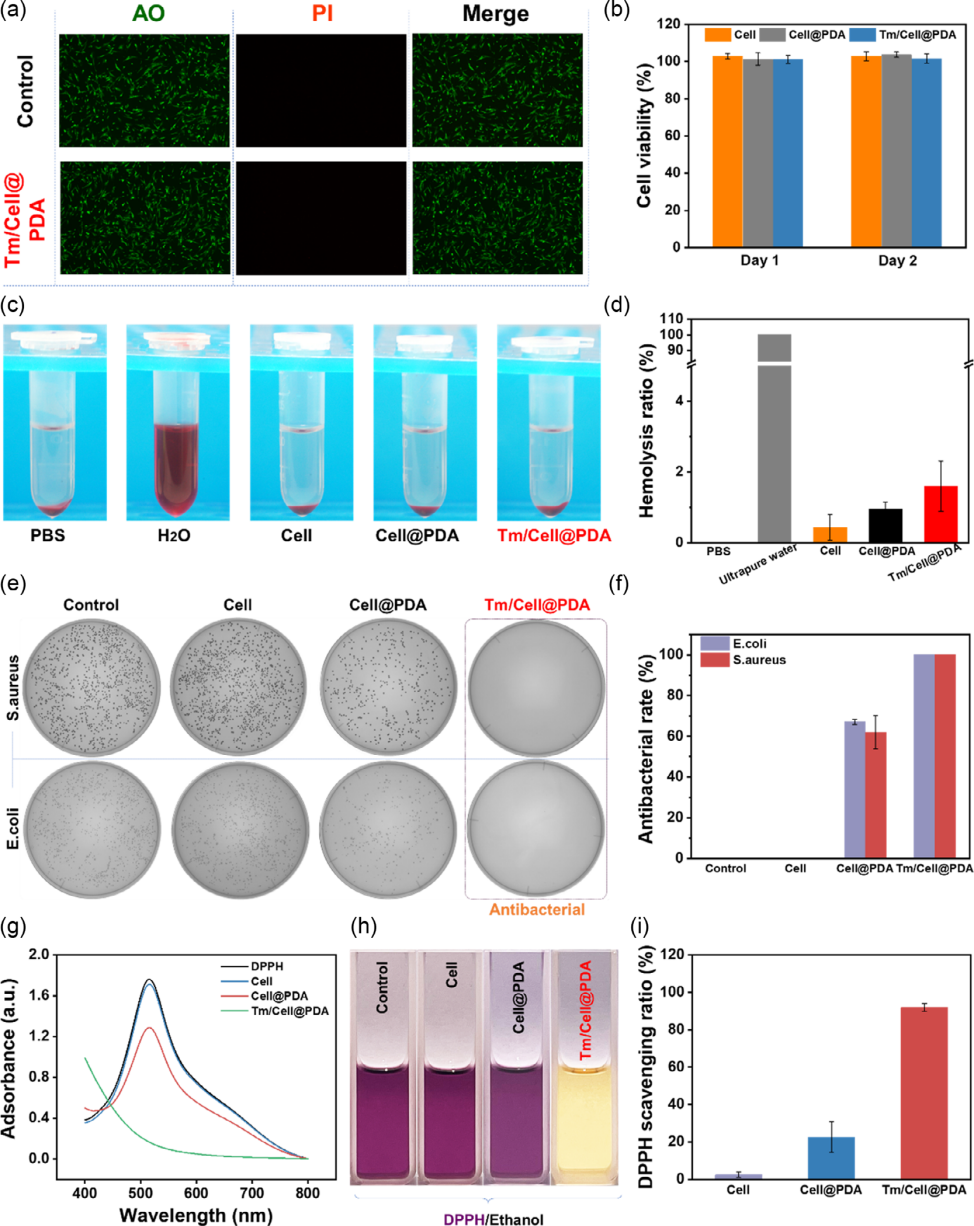
In vitro biological evaluation of the cellulose‐based cryogel microspheres. a) Dual‐fluorescence (AO/PI) viability of HFL1 cells cultured in Tm/Cell@PDA (20 mg mL^−1^) extracts using Ham's F‐12 K medium as the positive control. Scale bars, 500 μm. b) Cell viability of HFL1 cells incubated with the Cell, Cell@PDA, and Tm/Cell@PDA extracts at 24 h and 48 h (*n* = 4). c,d) Hemolysis analysis of the Cell, Cell@PDA, Tm/Cell@PDA extracts at different concentrations using ultrapure H_2_O as positive control and PBS as negative control (*n* = 4). e,f) Photographs of the bacterial colonies and the related anti‐bacterial ratios (*n* = 4) for 10 mg of Cell, Cell@PDA, and Tm/Cell@PDA co‐incubated with *E. coli* and *S. aureus* for 12 h, respectively, using PBS as the control group. g) UV‐Vis spectra of DPPH solution (ethanol) and DPPH solutions (ethanol) treated with Cell, Cell@PDA, Tm/Cell@PDA, respectively. h) Images of DPPH scavenging activity using different dressing materials. i) DPPH scavenging efficiency (*n* = 4) of different dressings co‐incubated with DPPH for 30 min; the DPPH group is the blank group. All data are presented as mean ± standard deviation (SD) according to duplicated experiments more than 3 times.

Biocompatibility, including cytocompatibility and hemocompatibility, is a prerequisite for applying cellulose‐based microspheres in irregular wound management. To examine the cytocompatibility of Cell, Cell@PDA, and Tm/Cell@PDA, series concentrations of microsphere extracts (20, 10, 5, 2.5, and 1.25 mg mL^−1^) were incubated with HFL1 cells and cytotoxicity was assessed by CCK‐8 assay. As shown in **Figure** **3**b, each group (20 mg mL^−1^) shows extremely high cell viability (over 99.5%) when the treatment time was extended from 24 to 48 h, demonstrating the excellent biocompatibility of all cellulose‐based cryogel microspheres. Moreover, each group with other different concentrations exhibited equally good cytocompatibility with 48 h of incubation (Figure S5, Supporting Information). A similar result was observed by double‐staining in the Tm/Cell@PDA group. Even at 20 mg mL^−1^, there were no dead cells (red color) observed after incubation for 48 h (Figure 3a). The Cell morphology was intact, indicating that Tm/Cell@PDA has excellent cytocompatibility and could be suitable for clinical use. Hemolysis testing showed that lysis of RBCs incubated in Cell, Cell@PDA, and Tm/Cell@PDA extracts (20, 10, 5, 2.5 and 1.25 mg mL^−1^; Figure S6, Supporting Information) was less than 2% (Figure 3d). The supernatants of all microsphere groups showed a similar degree of transparency as the control group (PBS), even at a high concentration (20 mg mL^−1^) (Figure 3c), indicating that the cellulose‐based cryogel microspheres have good hemocompatibility.

Bacterial infection hampers wound healing.^[^ ^40^ ^]^ Therefore, excellent antibacterial properties are essential for accelerating wound healing. The antibacterial performance of Cell, Cell@PDA, Tm/Cell@PDA against typical bacteria (e.g., *S. aureus* and *E. coli*) was evaluated by serial gradient dilution and colony counting. Compared to the control plates, the Cell group showed no antibacterial ability, while the Cell@PDA group showed limited antibacterial properties (62.0% against *S. aureus* and 67.1% against *E. coli*; Figure 3f). This result demonstrates that complete sterilization cannot be achieved by relying on the antimicrobial effect of PDA alone. The introduction of Tm yielded complete killing of *S. aureus* and *E. coli*, and the agar plates show no significant colony growth (Figure 3e,f). Thus, the synergistic antimicrobial effects of Tm (short‐term, highly effective antimicrobial agents) and PDA (long‐term, continuous protection) prevents early infection and promotes wound healing.

The initial stage of wound healing is usually accompanied by a strong inflammatory response, which induces ROS production, leading to intense oxidative stress, cellular damage, and delayed tissue regeneration and angiogenesis.^[^ ^41^ ^]^ Therefore, dressings with ROS‐scavenging ability can alleviate oxidative stress and promote wound healing. The antioxidant capacity of Cell, Cell@PDA, and Tm/Cell@PDA was investigated by studying DPPH radical scavenging assay. The absorption peaks almost overlapped for Cell group with the control group (only DPPH) and no evident color changes are observed in the Cell group compared with control group, indicating an absence of scavenging (Figure 3g,h). Cell@PDA group showed limited antioxidant capacity (22.7% of DPPH clearance) due to the presence of PDA. In contrast, Tm/Cell@PDA was endowed with excellent antioxidant capacity due to the synergistic effect Tm and PDA, and DPPH scavenging ratio was reached to 92.0% (Figure 3i). We thus conclude that Tm/Cell@PDA can scavenge excessive ROS at early phase to accelerate wound healing.

### 2.4 In Vivo Evaluation of the Pro‐wound Healing Performance of Cellulose‐based Microspheres

**Figure 4:**
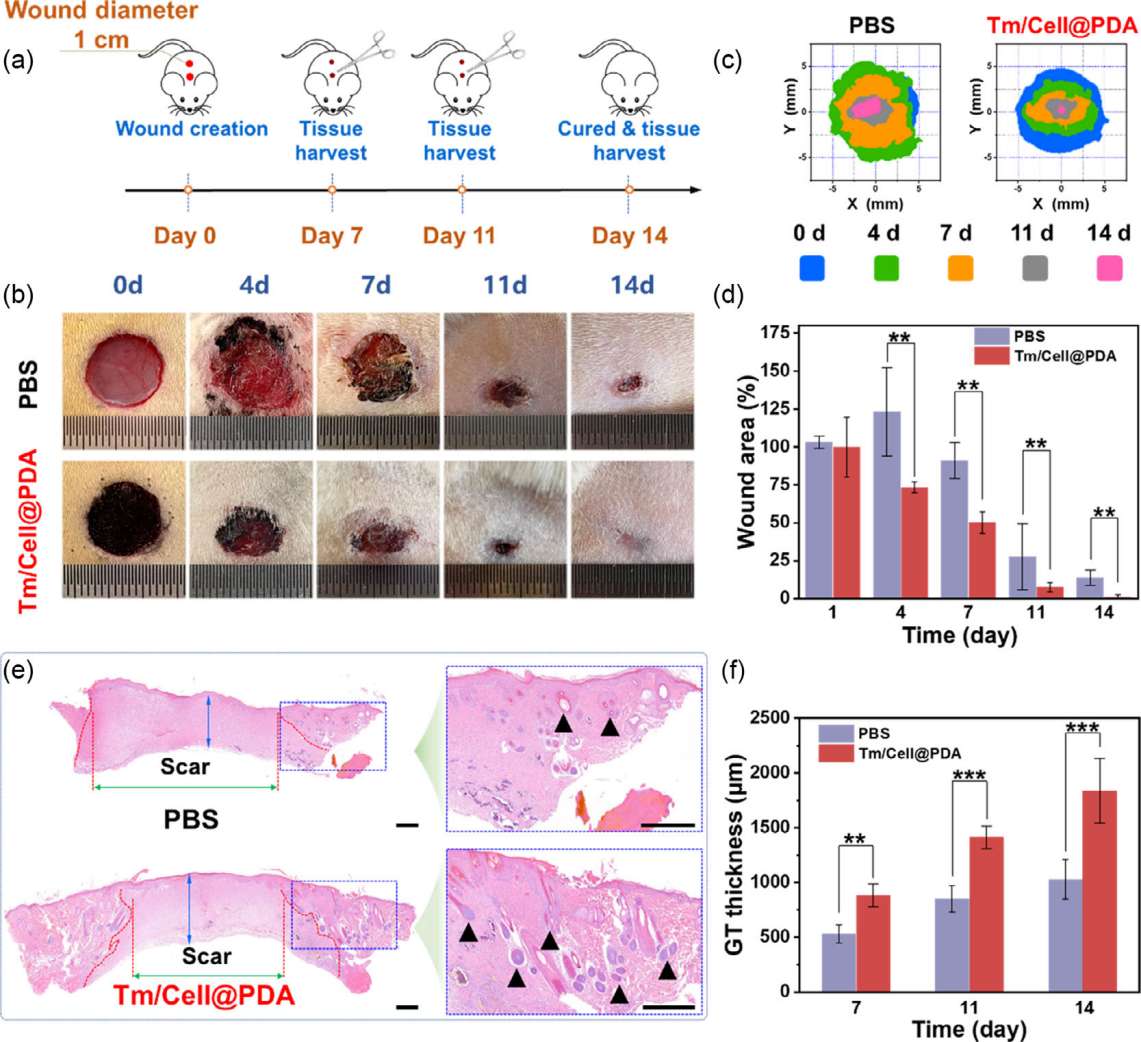
In vivo wound healing using cellulose‐based cryogel microspheres. a) Schematic procedures for evaluating the wound healing performance of dressing materials. b) Representative images of the full‐thickness skin defects on the back of rats treated with Tm/Cell@PDA for 14 d, using PBS as the control group. c) The wound‐bed closure process on rats treated with PBS and Tm/Cell@PDA. d) Quantitative wound area variations for each group on different days (*n* = 4). e) Regenerated tissue sections from the wounds on day 14 (Granulation tissue thickness, blue double‐headed arrows; scar width, green double‐headed arrows; regenerated hair follicle, black triangle) and the related (f) granulation tissue thickness (*n* = 4), scale bar, 500 μm. All data are presented as mean ± standard deviation (SD) according to duplicated experiments more than 3 times. *p* values for two tailed tests: **<0.01, ***<0.001.

As illustrated in **Figure** **4**a, the SD rat wound model is established and post‐treated by a series of specific procedures. Full‐thickness defect wounds (×2, Φ = 10 mm) are created on the back of rats with different treatments: 1) PBS, 2) Tm/Cell@PDA. Tm/Cell@PDA was evenly sprinkled on the wound, and the high hydrophilicity and rapid fluid absorption ability of the microspheres allowed them to absorb exudate and stably attach to the wound.

Wound healing was significantly faster with Tm/Cell@PDA than PBS (Figure 4b,c). For example, on the fourth day of treatment, due to the lack of bacterial growth inhibition, the wounds in the PBS group were enlarged and ulcerated, and the wound area reached 124.0%. In contrast, the wound area of the Tm/Cell@PDA group gradually decreased and began to form a scab, and no significant exudate was observed. On day 7, the wound area in the PBS group was still 91.1% (Figure 4d, *p* < 0.01), similar to the wound area at the beginning of healing, while the wound in the Tm/Cell@PDA group consistently showed an excellent healing trend with a wound area of only 49.7%, and the crusted surface completely covers the wound. By day 14, along with the growth of new tissue and the natural shedding of the scab, wounds treated with Tm/Cell@PDA group showed no visible defects, and the wound area shrunk dramatically to 1.1%, significantly less than the 13.8% in the PBS group (Figure 4d, *p* < 0.01). This apparent therapeutic difference can be attributed to the excellent antibacterial, antioxidant, and shape‐adaptive properties of Tm/Cell@PDA, providing a relatively sterile microenvironment to promote wound healing.

### 2.5 Histopathological and Immunofluorescence Evaluation of Cellulose‐Based Microspheres

**Figure 5:**
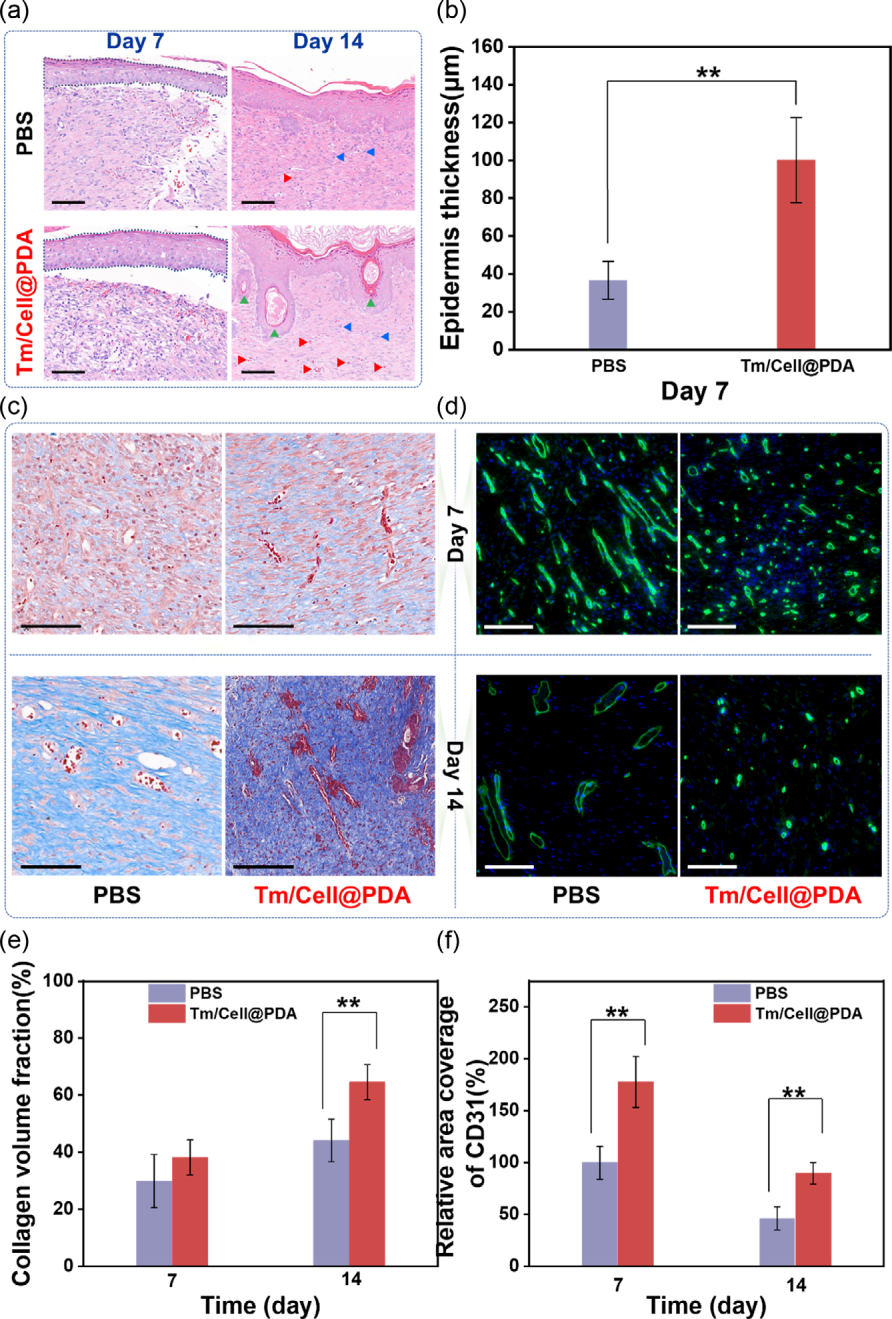
Histopathological and immunofluorescent analysis of the wounds treated with cellulose‐based cryogel microspheres. a) H&E and c) Masson's trichrome stained regenerated tissues from the wounds on day 7 and day 14 (new epidermis, blue dash lines; blood vessel, red triangle; fibroblast, blue triangle; and hair follicle, green arrow; scale bar, 100 μm) and the related b) epidermis thickness (*n* = 4) and e) collagen volume fraction (*n* = 4). d) The immunofluorescent images of CD31 stained regenerated tissues on day 7 and day 14, and f) the relative area coverage of CD31 (*n* = 4). Scale bar, 100 μm. Cell nuclei, blue fluorescence (DAPI); blood vessels, green fluorescence (CD31). All data are presented as mean ± standard deviation (SD) according to duplicated experiments more than 3 times*. p* values for two tailed tests: **<0.01.

Histopathological staining and immunofluorescence experiments were used to evaluate wound shrinkage and tissue regeneration. On day 14, tissue sections stained by H&E showed thicker granulation tissue in the Tm/Cell@PDA group (vertical double‐head arrow, 1837.7 ± 294.8 μm; Figure 4e,f) and a narrower scar width (horizontal double‐head arrow, 2473.2 ± 392.7 μm; Figure 4e and S7, Supporting Information) than in the PBS group (granulation tissue, 1028.8 ± 179.8 μm; scar width, 4567.5 ± 466.6 μm). Furthermore, more new hair follicles (black triangles) were formed, confirming the pro‐wound contraction potential of Tm/Cell@PDA. With the sprouting of granulation tissue and the disappearance of the scar, the new epidermis gradually regenerates. The regenerating epidermis of the wound tissue on day 7 grew more ideally in the Tm/Cell@PDA group (100.1 ± 22.8 μm) than in the PBS group (36.7 ± 10.0 μm, *p* < 0.01) (**Figure** **5**a,b). A large number of well‐developed hair follicles (green arrows), fibroblasts (blue arrows), and blood vessels (red arrows) were observed in the repaired area on day 14. Masson trichrome staining showed the percent of collagen in the healing wounds (Figure 5c, blue area). Tm/Cell@PDA‐treated wounds had a higher density of collagen deposition (day 7, 44.2% ± 7.5%; day 14, 64.6% ± 6.1%) compared to the PBS control (Figure 5c,e). Furthermore, the regenerated tissue structures in Tm/Cell@PDA group are relatively intact.

Neovascularization is a direct indicator to reflect the angiogenic capacity of the dressing.^[^ ^42^, ^43^ ^]^ The efficacy of Tm/Cell@PDA in promoting angiogenesis was evaluated by detecting neovascularization marker CD31. Capillary density was higher in tissues treated with Tm/Cell@PDA (greater coverage of CD31‐positive markers, 178%), indicating accelerated angiogenesis and wound healing (Figure 5d,f). Histopathological and immunofluorescence analyses conclusively demonstrate the excellent pro‐healing effect of Tm/Cell@PDA.

In short, the rapid water absorption, and synergistic antibacterial and antioxidant properties of Tm/Cell@PDA contribute to accelerating the healing process. In the early stages of wound healing, exudate is rapidly absorbed and Tm is quickly released to inhibit bacterial infection and eliminate excessive ROS. After the complete release of Tm, PDA acts as the last line of defense to hinder bacterial proliferation and accelerate wound healing.

## 3 Conclusion

In summary, we have developed cellulose‐based microspheres (Tm/Cell@PDA) with multiple functions (antioxidant, antibacterial, hygroscopic and wet‐adhesion) that act as adaptive‐shaped dressings to address the limitations of irregular wound healing. The tiny size, high water absorption, and hydrophilicity of Tm/Cell@PDA allow them to adsorb wound exudate quickly and firmly adhere to the wound without external force. Antimicrobial ingredient (Tm) is completely released within a few hours, alleviating oxidative stress and avoiding infection of bacteria in the early stages of healing. PDA provides long‐term protection due to their contact‐active antimicrobial mechanism. In vitro and in vivo assays demonstrated that the multifunctional cryogel microspheres Tm/Cell@PDA provide excellent pro‐healing performance by promoting the growth of granulation and neovascularization. Thus, Tm/Cell@PDA provides an effective, promising, and practical combined therapy for treating irregular wounds, which may bring new hope for clinical application.

## 4 Experimental Section

4.1

4.1.1

##### Materials

Microcrystalline cellulose (MCC) with an average degree of polymerization (DP) of 220 is purchased from Beijing Fengli Jingqiu Pharmaceutical Co., Ltd. (China). Dopamine hydrochloride (DA), thymol (Tm), tert‐butanol (TBA), and tris hydrochloride (Tris‐HCl) are commercially available from Innochem (Beijing). 1‐allyl‐3‐methylimidazolium chloride (AmimCl) is supplied by Henglian New Materials Co., Ltd. (Shandong, China). AO/PI staining solution, Phosphate Buffer Saline (PBS), Ham's F‐12K (Kaighn's) medium, fetal bovine serum (FBS), penicillin/streptomycin solution (P/S), trypsin‐EDTA digestion solution, CCK‐8 kit are supplied by Biorigin (Beijing). 10% natural buffered Formalin solution is purchased from Leagene Biotechnology (Beijing). MCC is dried with a vacuum oven at 80 °C for 24 h before use. Other chemical reagents and solvents are used directly without purification.

##### Preparation of Cellulose Hydrogel Microspheres (Cell‐hm)

Microcrystalline cellulose (15.0 g) is mixed with AmimCl (135.0 g) in a three‐neck flask and stirred continuously for 2 h at 80 °C under vacuum to obtain a homogeneous solution of cellulose (10 wt%). DMF (150.0 g) is added and stirred to obtain a diluted cellulose solution (5 wt%).

Cell‐hm are prepared using a modified coaxial airflow method. A plastic syringe tube (100 mL) with a Luer fitting is filled with the 5 wt% cellulose solution. To this is attached a coaxial ejector consisting of an inner nozzle (inner diameter, Φ_1_ = 210 μm; outer diameter, Φ_2_ = 410 μm) for the cellulose solution and an outer nozzle (Φ_3_ = 1.20 mm) for compressed air is attached to the syringe tube by Luer fitting. The cellulose solution is extruded under 0.1 MPa and sheared into droplets by high‐speed compressed air (4.5 L min^−1^). Finally, Cell‐hm are generated after the droplets fly into the coagulation bath (ethanol).

##### Preparation of Cellulose@polydopamine Hydrogel Microspheres (Cell@PDA‐hm)

Cell@PDA‐hm are prepared by in‐situ polymerization of dopamine (DA) on Cell‐hm. Cell (60 g) is dispersed in 600 mL of H_2_O, and the pH is adjusted to 8.4 ± 0.1 using Tris‐HCl. DA (1.2 g) is added, and the Cell dispersion is gently stirred at 30 °C for 24 h. Finally, unreacted reagents are removed by rinsing with water, yielding black–brown Cell@PDA‐hm.

##### Preparation of Thymol/Cellulose@polydopamine Hydrogel Microspheres (Tm/Cell@PDA‐hm)

Cell@PDA‐hm (60 g) are soaked in 50 wt% tertiary butanol/water (TBA/H_2_O) for 24 h, replacing the liquid three times every 8 h. Tm (0.4 g) is added and completely dissolved. The solution is gently stirred at room temperature for 24 h to obtain Tm/Cell@PDA‐hm.

##### Preparation of Cellulose Cryogel Microspheres (Cell), Cellulose@polydopamine Cryogel Microspheres (Cell@PDA), Thymol/Cellulose@polydopamine Cryogel Microspheres (Tm/Cell@PDA)

60 g of Cell‐hm and Cell@PDA‐hm are soaked in 50 wt% tertiary butanol/water (TBA/H_2_O) for 24 h, replacing the liquid three times every 8 h. Subsequently, Cell, Cell@PDA, Tm/Cell@PDA are obtained by freeze‐drying of Cell‐hm, Cell@PDA‐hm, Tm/Cell@PDA‐hm.

##### Micromorphological Analysis

Micromorphologies of the microspheres are observed using a field emission‐scanning electron microscope (FE‐SEM, JEOL JSM‐6700F), and the EDS analysis is performed with an FE‐SEM with energy dispersive spectroscopy (Hitachi S‐8020). All microspheres are sputter‐coated with gold for 180 s before observation. Particle size of the microspheres are respectively measured with a laser particle size analyzer (Bettersize 2600). The N_2_ adsorption‐desorption isotherms for the specific surface area of the microspheres are carried out on a Quantachrome instrument (NOVA3200e and iQ, 77 K). All microspheres are vacuumed at 100 °C for 12 h to degas before the tests. Elemental analysis is implemented with an XPS spectrometer (Thermo Fisher ESCALAB 250 Xi). ATR‐FTIR spectra (650–4000 cm^−1^, resolution/4 cm^−1^, scans number/64) are measured by using the Thermo Nicolet 6700 Fourier transform infrared spectrometer (Thermo Fisher, USA).

Powder X‐ray diffraction is recorded in reflection mode in the angular range of 5°–50° (2*θ*) with a scanning speed of 5° min^−1^ by using an X‐ray diffractometer (PANalytical, Netherlands), and the wavelength of the Cu Kα radiation source is 1.54 Å. The samples are ground to fine powders and then dried at room temperature to prepare isotopically oriented powder for X‐ray diffraction measurement.

The UV‐vis spectra of the thymol of microspheres (100 mg; Cell, Cell@PDA, Tm/Cell@PDA) soaked in PBS (60 mL) for different times (3 min, 3 h) are recorded with a Shimadzu UV 2600 system.

##### PBS Absorption Capacity

PBS absorption ratio (%) of the microspheres is measured according to the method reported in the literature as described.^[^ ^44^ ^]^ The dry microspheres (*M*_dry_) are thoroughly immersed in PBS for 10 s, 2 min, then the wet microspheres (*M*_wet_) are removed, and the excess PBS drained with filter paper. Measurements are performed three times for each sample. PBS absorption ratio (%) is given by the formula

(2) $$\text{PBS adsorption ratio }\left(\%\right)=\left(M_{\text{wet}}-M_{\text{dry}}\right)/M_{\text{dry}}\times 100\%$$

##### Release Kinetics of Thymol in Tm/Cell@PDA

The release kinetics of Tm in Tm/Cell@PDA was investigated using a UV‐vis calibration curve. The UV‐vis spectra of a series concentration of Tm/ethanol solutions and the Tm in Tm/Cell@PDA dissolved in PBS were recorded with a Shimadzu UV 2600 system. The concentration versus absorbance calibration curve of Tm dissolved in ethanol was measured at 276 nm. Tm/Cell@PDA (100 mg) were soaked in 60 mL of PBS (pH = 7.4), and the release of Tm was monitored by absorbance at 276 nm. Finally, the kinetic release plot of Tm was calculated according to the concentration versus absorbance calibration curve.

##### Determination of Thymol Content in Tm/Cell and Tm/Cell@PDA

The UV‐vis absorption spectra of Tm were obtained from a series of Tm–ethanol solutions, and standard curves (Abs‐Concentration) were established by the linear relationship between its characteristic absorption value at 276 nm and its concentration. Tm in Tm/Cell and Tm/Cell@PDA was extracted using a Soxhlet extractor, and the total amount and actual release of Tm were assessed. Microspheres (200 mg) were placed in a Soxhlet extractor, 500 mL ethanol was added and heated to reflux more than 20 times at 90 °C to ensure complete elution of Tm. The *λ*_max_ of the solution at 276 nm was measured by UV spectrophotometry and substituted into the Abs‐Concentration standard curve to obtain the total amount of Tm. Microspheres (200 mg) were placed in PBS (500 mL) and immersed for 24 h at 150 rpm min^−1^ on a shaker. The amount of Tm release (*λ*_max_) was measured at 276 nm and calculated from the Abs‐Concentration calibration curve. Each assessment was performed three times.

##### Cytocompatibility

A CCK‐8 assay was used to evaluate the cytotoxicity of Cell, Cell@PDA, and Tm/Cell@PDA in HFL1 cells (human fetal lung fibroblast Cell line). The cells were seeded (8 × 10^3^ cells/well) in Ham's F‐12 K medium (containing 10% FBS and 1% P/S) in a 96‐well microtiter plate (200 μL per well) and incubated at 37 °C in 5% CO_2_ atmosphere (duplicated samples, *n* = 4). Samples (20, 10, 5, 2.5, and 1.25 mg mL^−1^) were extracted by soaking in the medium for 24 h. Fresh medium exchange was performed at 24‐ and 48‐h of incubation; then, 10 μL of CCK‐8 was added to each well and incubated for 3 h at 37 °C. Absorbance for each cell was measured at 450 nm using a microplate reader (CLARIOstar Plus, BMG Labtech). Medium alone was used as the control. Cell viability (%) was calculated as follows

(3) $$\text{Cell Viability }\left(\%\right)={\text{ Abs}}_{\text{sample}}/{\text{Abs}}_{\text{control}}\times 100\%$$

##### Dual‐Fluorescence Cell Viability Assay

Dual‐fluorescence cell viability using acridine orange (AO) and propidium iodide (PI) was used to assess cytocompatibility of Tm/Cell@PDA in HFL1 cells. Cells were seeded (2 × 10^4^ cells/well) in 1000 μL Ham's F‐12 K medium in a 24‐well plate and incubated at 37 °C in a 5% CO_2_ atmosphere for 24 h (duplicated samples, *n* = 3). The medium was replaced with Tm/Cell@PDA (20 mg mL^−1^) and incubated for another 24 h (37 °C, 5% CO_2_). The medium was removed and replaced with 500 μL fresh medium and 50 μL AO/PI staining solution, then incubated in the dark for 15 min. After a final PBS rinse, the live/dead HFL1 cells were observed under an inverted microscope (Nikon Ti2‐U). Medium alone was used as the control.

##### Hemocompatibility Tests

Hemocompatibility of Cell, Cell@PDA, and Tm/Cell@PDA was measured as a hemolysis ratio. The extracts of samples were prepared by PBS immersing at 37 °C for 24 h and then diluted in a concentration gradient (20, 10, 5, 2.5, 1.25 mg mL^−1^). A suspension of erythrocytes (6 vol%) in PBS was obtained from fresh citrate‐anticoagulated rat whole blood (3.8% sodium citrate/blood, 1/9 v v^−1^) by centrifuging and washing in PBS three times (3000 rpm, 10 min). The suspension of erythrocytes and extracts were incubated (1:1) at 37 °C for 3 h, then centrifuged at 3000 rpm for 10 min (duplicated samples, *n* = 4). The supernatants were measured at 540 nm with a microplate reader. Ultrapure H_2_O and PBS were used as positive (+) and negative (−) controls, respectively. The hemolysis ratio (%) is calculated as follows

(4) $$\text{Hemolysis ratio }\left(\%\right)=\left({\text{Abs}}_{\text{sample}}-{\text{Abs}}_{\left(-\right)}\right)/\left({\text{Abs}}_{\left(+\right)}-{\text{Abs}}_{\left(-\right)}\right)\;\times 100\%$$

##### In Vitro Antibacterial Activity

Gram‐positive (S. aureus) and Gram‐negative bacteria (E. coli) were used to assess the antibacterial activity of Cell, Cell@PDA, and Tm/Cell@PDA. In a 96‐well microtiter plate, 10 μL bacterial suspension (OD_S.aureus_ = 1.0, OD_E.coli_ = 0.6) and 100 mg of Cell, Cell@PDA, and Tm/Cell@PDA were incubated for 30 min in the dark. The bacterial suspensions were serially diluted in PBS to (1 × 10^−4^) and 100 μL was spread on semi‐solid agar. Colonies were counted after incubating for 16 h at 37 °C. Colony forming units (CFU) of control (bacterial solution in PBS without cellulose cryogel microspheres) and experimental groups were used to calculate the antibacterial ratio (%) as follows

(5) $$\text{Antibacterial ratio }\left(\%\right)=\left({\text{CFU}}_{\text{control}}-{\text{CFU}}_{\text{sample}}\right)/{\text{CFU}}_{\text{control}}\times 100\%$$

##### Antioxidant Capacity

The antioxidant capacity of Cell, Cell@PDA, and Tm/Cell@PDA was defined as DPPH radical scavenging ratio (%). DPPH (80 μM, 4 mL) in ethanol plus 100 mg of Cell, Cell@PDA, and Tm/Cell@PDA were incubated in the dark for 30 min, then measured at 517 nm using a UV‐vis spectrophotometer. Each experiment was performed in replicates of four. The DPPH scavenging ratio was calculated as follows

(6) $$\text{DPPH scavenging ratio }\left(\%\right)=\left[1-\frac{A_{s}}{A_{c}}\right]\times 100\%$$

where *A*_s_ is the absorbance of the microsphere solution and *A*_c_ is the absorbance of DPPH ethanol alone.

##### In Vivo Wound Healing

Adult male Sprague‐Dawley (SD) rats (150–170 g) are supplied by Vital River Laboratory Animal Technology Co., Ltd (Beijing). All experiments were approved by the Animal Care and Use Committee of General Hospital of Chinese People's Liberation Army (Protocol No. *2023‐X19‐17*). Full skin wounds (×2; diameter, 10 mm) were made on the rat's backs, then treated with 5 mg Tm/Cell@PDA or PBS (control) (duplicated experiments, 24 SD rats, *n* = 12 per group). The wound‐healing process (0–14 d) was recorded using a camera and a ruler for reference. Wound‐healing rate was measured by tracing the wound area on plotting paper.

##### Histopathology & Immunofluorescence Analysis

Rats were sacrificed for histopathology and immunofluorescence analysis at 7, 11, and 14 d after wound treatment. Tissues sampled were fixed in 10% natural buffered Formalin and gradually dehydrated with the graded ethanol (70%, 80%, 90%, and 100%), following embedment in paraffin. Tissue slices were stained with hematoxylin and eosin (H&E), Masson trichrome, and CD31. The staining results are analyzed using the software CaseViewer 2.0. Histopathology was performed by a blinded pathologist to assess the inflammatory response, fibrosis, and neovascularization.

##### Statistical Analysis

All data are presented as mean ± standard deviation (SD) according to duplicated experiments more than 3 times. The two‐tailed *t*‐test is used to determine the statistical significance between the two groups and *p* < 0.05 was considered statistically significant. *p* values for two tailed tests: *<0.05, **<0.01, ***<0.001, ****<0.0001.

## Conflict of Interest

The authors declare no conflict of interest.

## Supporting information

Supplementary Material

## Data Availability

The data that support the findings of this study are available from the corresponding author upon reasonable request.
