# Supplementary material for: Surface Wrinkled Microsphere Enhanced Irregular Wound Healing Through Synergistic Hygroscopicity, Reversible Wet‐Adhesion and Antibacterial Properties
Source: Small Sci. 2024 Jan 3;4(2):2300216. doi: 10.1002/smsc.202300216 (PMC11934996; doi:10.1002/smsc.202300216)
Supplement: Supplementary file 1 — Supplementary Material [file SMSC-4-2300216-s001.pdf]

Supporting Information

Surface wrinkled microsphere enhanced irregular wound healing through synergistic hygroscopicity, reversible wet-adhesion and antibacterial properties

Zhan Xu [a,b], Yuqian Cui [a,b], Weiguo Tian [a,*], Feifei Sun [c,*], Jun zhang [a,b,]

[a] *Beijing National Laboratory for Molecular Sciences, CAS Key Laboratory of Engineering Plastics, Institute of Chemistry Chinese Academy of Sciences (CAS), Beijing 100190, China;*

[b] *University of Chinese Academy of Sciences, Beijing 100049, China.*

[c] *CAS Key Laboratory for Biomedical Effects of Nanomaterials & Nanosafety, CAS Center for Excellence in Nanoscience, National Center for Nanoscience and Technology, Beijing, 100190, China*

[*] *E-mail: wgtian@iccas.ac.cn; sunff2022@nanoctr.cn;*

Content

1. Supplementary Tables: Table S1

2. Supplementary Figures: Figure S1 to S7

Supplementary Tables

Table S1 Fundamental parameters of the Cell, Cell@PDA, Tm/Cell@PDA.

Sample	Specific surface area<br>m^2/g	Average pore size<br>nm
Cell	173.79	24.98
Cell@PDA	154.12	15.73
Tm/Cell@PDA	157.85	16.18

Supplementary Figures

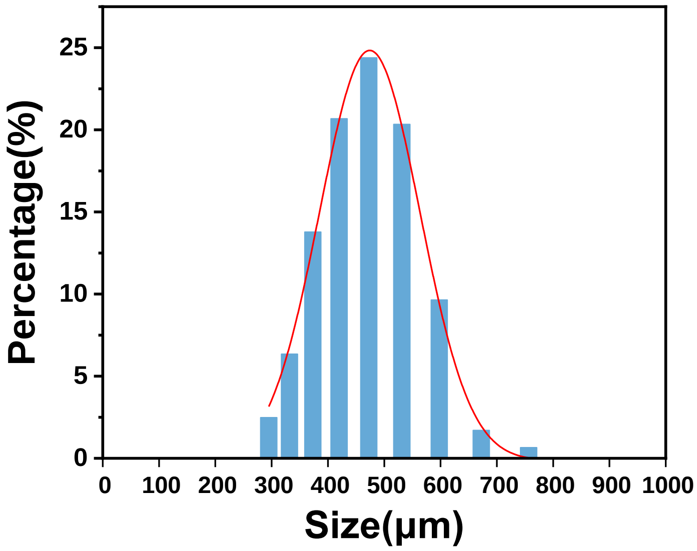

Figure S1. Diameter distribution of cryogel microspheres Tm/Cell@PDA.

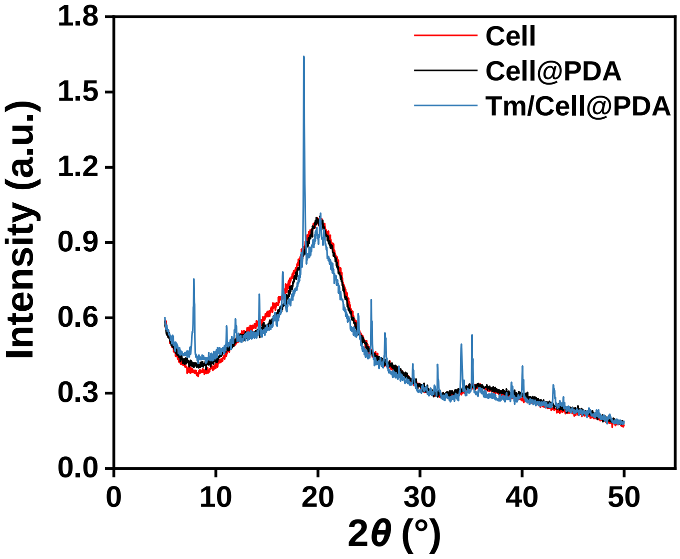

Figure S2. X-ray diffraction of Cell, Cell@PDA, Tm/Cell@PDA.

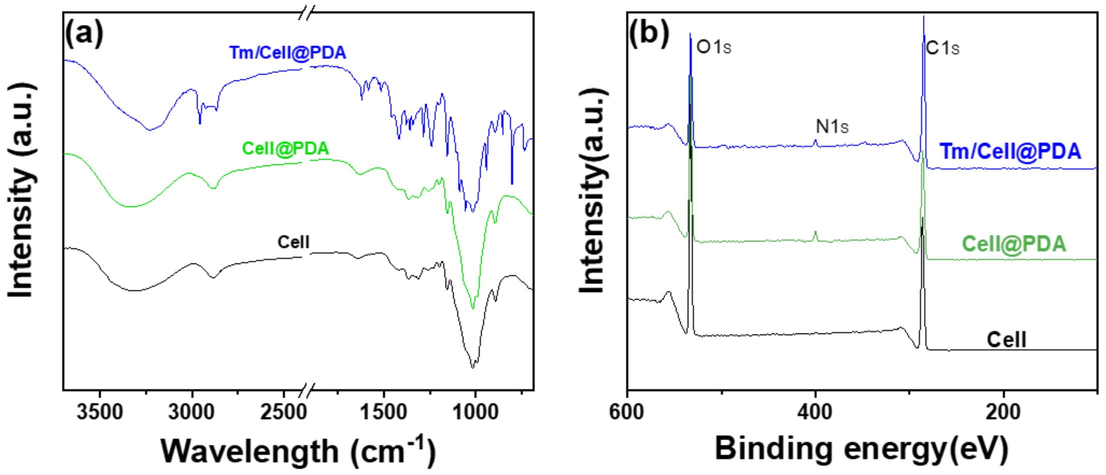

Figure S3. (a) FTIR, **(b)** XPS spectra of cellulose-based microspheres.

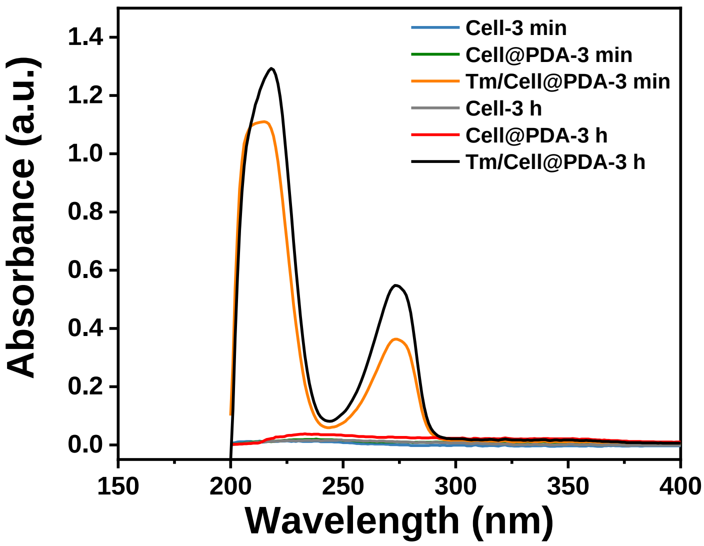

Figure S4. UV-vis spectra of thymol released from Cell, Cell@PDA, Tm/Cell@PDA immersed in PBS (pH = 7.4) for different times.

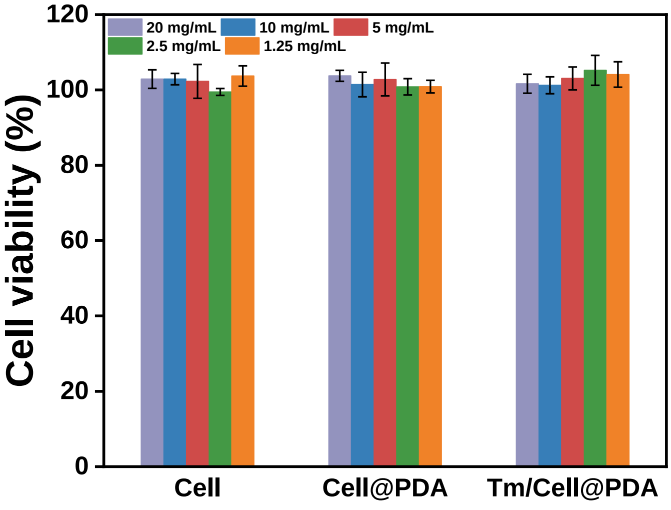

Figure S5. Cell viability of HaCaT cells incubated with the Cell, Cell@PDA, Tm/Cell@PDA extracts at different concentrations (n = 4).

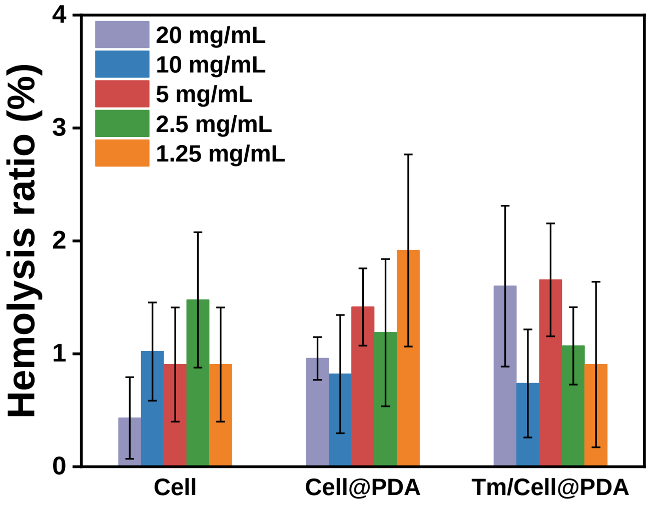

Figure S6. Hemolysis assay of the extracts obtained by socking Cell, Cell@PDA, Tm/Cell@PDA in PBS at different mass concentrations, using ultrapure H_2O as positive control and PBS as negative control (n = 4).

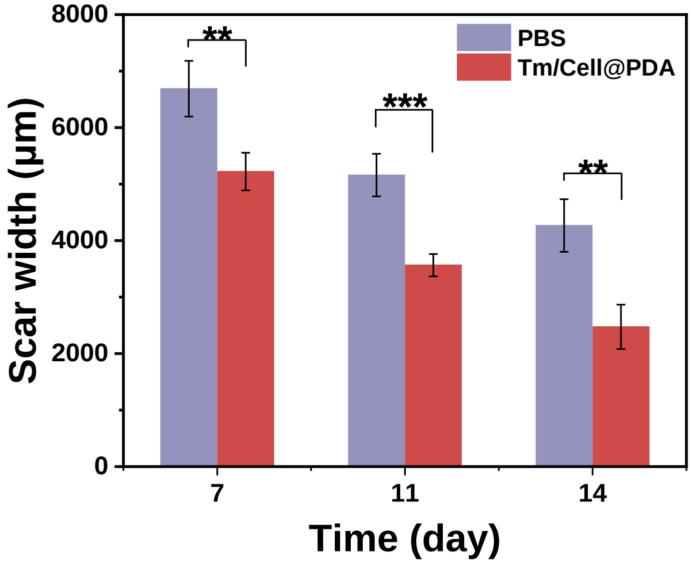

Figure S7. Scar width of wounds treated with Tm/Cell@PDA for different times, using PBS as control group (n = 4).
